# Enhanced CT-based deep learning radiomics for bladder cancer prognosis

**DOI:** 10.1016/j.mmr.2026.100065

**Published:** 2026-08-22

**Authors:** William C. Cho

**Affiliations:** Department of Clinical Oncology, Queen Elizabeth Hospital, Hong Kong SAR, China

**Keywords:** Artificial intelligence (AI), Computed tomography (CT), Deep learning (DL), Muscle-invasive bladder cancer, Radiomics

This commentary refers to the article available online at https://www.sciencedirect.com/science/article/pii/S2054936926000014

Bladder cancer (BLCA) is the eighth most frequent cancer and the thirteenth leading cause of cancer death worldwide, with about 635,000 new cases and 228,000 deaths estimated in 2024 [1]. The clinical management of BLCA relies on one key question: has the tumor invaded the muscularis propria? This binary distinction, non-muscle-invasive vs. muscle-invasive binary classification, decides whether a patient undergoes organ-preserving endoscopic resection or radical cystectomy with systemic therapy. Yet the current gold standard, histopathological evaluation of transurethral resection specimens, has its constraints [2]. A biopsy may not capture the full extent of the tumor. The heat from transurethral resection equipment can distort tissue architecture. Variability among operators introduces inconsistency. These issues lead to understaging in many cases, with incidence varying by institution and patient population [3]. He *et al.* [4] have developed a robust and validated artificial intelligence (AI)-driven solution to this longstanding clinical challenge.

Their work stands out for its methodological approach. The authors combined two complementary methods, traditional radiomics (which collects measurable features such as tumor size, shape, and interface characteristics) and deep learning (DL), particularly a convolutional neural network, which captures complex and high-dimensional representations that traditional feature extraction often overlooks [4], [5]. This hybrid system resembles how an experienced clinician weighs measurable tumor characteristics against intuitive pattern-based assessment gained from years of practice. Internal validation showed an area under the receiver operating characteristic curve (AUC) of 0.807, while the external multi-center cohort achieved 0.783. These outcomes significantly better than those of the clinical model used alone. They also exceeded the predictive abilities of either the radiomic or DL components when used separately [5]. Nevertheless, several limitations warrant careful consideration. The DL backbone (ResNet101) was initially developed for natural image recognition. While its transferability is impressive, it is not fully optimized for computed tomography (CT) imaging. Future improvements will likely rely on designs specifically tailored for medical use. Some methodological points deserve consideration as well. The validation framework relies on a single training/validation split without cross-validation or bootstrap resampling. Performance metrics derived from a single split are highly sensitive to how that split was drawn. Without repeated sampling, there is no estimate of variance. On the one hand, the use of generative adversarial network (GAN)-based super-resolution also warrants close examination. GAN creates synthetic images that do not correspond to any actual patient scan. The boundary between synthetic and real data in the validation set is not clearly stated. If GAN-generated images are present in the validation set, reported performance will be inflated. On the other hand, the intensity standardization across centers used a fixed Hounsfield unit (HU) range of −75 to 175. This lower bound partially excludes perivesical fat signal, which typically occupies −50 to −100 HU. Given that perivesical fat plane integrity is a key criterion for T3 staging, this truncation may limit the model’s sensitivity to invasion-related features. The study’s reliance on CT (the primary imaging modality for 2249 of 2909 patients) reflects clinical reality, yet CT’s limited ability to resolve soft tissue compared to magnetic resonance imaging remains a fundamental limitation. The generalizability of the study population deserves attention. Most training and validation cohorts came from Chinese academic centers, representing mostly East Asian patients. The inclusion of The Cancer Genome Atlas (TCGA)-BLCA provided a preliminary test of cross-ethnic applicability, but more extensive validation in large, diverse international cohorts is necessary. The performance drop observed with TCGA-BLCA data (AUC=0.519) highlights the fragility of models when applying to different ethnic groups, imaging protocols, and clinical workflows. Strategies for adapting to domain differences and including multi-regional cohorts will be needed to enhance robustness.

Particularly encouraging is the model’s ability to generalize across multiple institutions. Poor generalizability often challenges AI applications in medical imaging [6]. Against this backdrop, the authors’ efforts to tackle data variability are commendable. Their use of GAN-based super-resolution, along with careful feature harmonization, serves as a useful methodological reference for future multi-center studies. The prospective human-AI interaction experiment, however, offers perhaps the most relevant clinical insight. AI assistance improved diagnostic accuracy among urologists, especially junior ones, addressing a real-world challenge where specialized uroradiology expertise is unavailable. The significant increase in sensitivity is particularly important. Missing a muscle-invasive BLCA diagnosis can lead to delayed treatment and disease progression, while over-calling may result in aggressive but potentially unnecessary surgery [7].

Beyond immediate applications, this methodological approach could be extended to other key questions in BLCA treatment. Predicting responses to neoadjuvant chemotherapy, forecasting immunotherapy outcomes, identifying patients at high risk for recurrence, and anticipating treatment resistance before it becomes obvious are all possible areas for study [8]. While the authors mentioned applicability to other malignancies, BLCA itself deserves priority since the infrastructure and expertise developed here can be directly utilized for these related tasks. Cervical cancer represents another cancer type with a comparable clinical need for non-invasive risk stratification. Notably, CT imaging is already widely integrated into its standard care.

Integrating such AI tools into multidisciplinary tumor boards represents a natural step forward. Other studies have already started combining imaging-based risk assessments with molecular analysis (genomic, transcriptomic, or histopathological data) to refine treatment decisions [9], [10]. These multimodal approaches, which integrate DL signatures with molecular biomarkers, offer a more complete picture of tumor biology and patient outcomes. The DL radiomics nomogram risk score, incorporated as a factor in a multivariate Cox model alongside clinical factors, shows independent prognostic value. This suggests the imaging characteristics reflect not only invasion depth but also the underlying aggressiveness of the tumor.

Several specific considerations merit emphasis for clinicians evaluating this model’s potential adoption. The authors did not stratify their analysis by patient subgroups that might derive particular benefit from AI-assisted interpretation, such as those with ambiguous imaging findings, equivocal biopsy results, or lesions at the extremes of size where visual assessment is most challenging. This represents a missed opportunity to identify the specific clinical scenarios where the model adds the greatest value. Similarly, the study did not separately analyze patients with prior intravesical therapy, variant histology, or multifocal disease. These subgroups are over-represented in real-world practice, and understanding model performance within them would substantially enhance clinical translation. The question of comparative performance against existing prediction models also remains incompletely addressed. While the authors validated their model on an external dataset, they did not benchmark it directly against previously published radiomic signatures or the vesical imaging-reporting and data system (VI-RADS) scoring in the same patient cohort. Without head-to-head comparison, clinicians cannot easily determine whether this hybrid approach offers meaningful advantages over simpler, more established methods. The authors’ assertion that their model outperforms prior work rests largely on indirect comparisons across disparate populations with varying inclusion criteria, which must be interpreted with caution.

In conclusion, He *et al.* [4] have developed a multi-center-validated model that improves diagnostic accuracy for muscle invasion and prognostic evaluation for overall survival. It gives clinicians a non-invasive tool to navigate the complex treatment landscape of BLCA. The next challenge is to translate these promising results into routine clinical practice. This requires implementation studies, health-economic assessments, and, above all, building trust within the clinical community. The journey from algorithm to bedside is rarely simple, but studies like this one indicate a clear path forward.

## Abbreviations

AI: Artificial intelligence
AUC: Area under the receiver operating characteristic curve
BLCA: Bladder cancer
CT: Computed tomography
DL: Deep learning
GAN: Generative adversarial network
HU: Hounsfield unit
TCGA: The Cancer Genome Atlas

## Funding

Not applicable.

## Data Availability

Not applicable.
